# Anticoagulation in critically ill patients on mechanical ventilation suffering from COVID-19 disease, The ANTI-CO trial: A structured summary of a study protocol for a randomised controlled trial

**DOI:** 10.1186/s13063-020-04689-1

**Published:** 2020-09-07

**Authors:** Nadir Kharma, Stefan Roehrig, Ahmed Atef Shible, Moustafa Sayed Elshafei, Dema Osman, Ingi Mohamed Elsaid, Salma Faisal Mustafa, Asjad Aldabi, Osamah A.M. Smain, Marcus D. Lance

**Affiliations:** 1 Department of Medical Intensive Care, Hamad Medical Corporation (HMC), Al-Rayyan Road, Doha, Qatar; 2 Department of Anesthesiology, Intensive Care and Perioperative Medicine, Hamad Medical Corporation (HMC), Al-Rayyan Road, Doha, Qatar; 3 Department of Pharmacy, Hamad Medical Corporation (HMC), Al-Rayyan Road, Doha, Qatar; 4 Department of Medical Education, Hamad Medical Corporation (HMC), Al-Rayyan Road, Doha, Qatar

**Keywords:** COVID-19, randomised controlled trial, protocol, anticoagulation, thrombosis, Anti-Co trial

## Abstract

**Objectives:**

To assess the effect of anticoagulation with bivalirudin administered intravenously on gas-exchange in patients with COVID-19 and respiratory failure using invasive mechanical ventilation.

**Trial design:**

This is a single centre parallel group, superiority, randomized (1:1 allocation ratio) controlled trial.

**Participants:**

All patients admitted to the Hamad Medical Corporation -ICU in Qatar for COVID-19 associated respiratory distress and in need of mechanical ventilation are screened for eligibility.

Inclusion criteria: all adult patients admitted to the ICU who test positive for COVID-19 by PCR-test and in need for mechanical ventilation are eligible for inclusion. Upon crossing the limit of D-dimers (1.2 mg/L) these patients are routinely treated with an increased dose of anticoagulant according to our local protocol. This will be the start of randomization.

Exclusion criteria: pregnancy, allergic to the drug, inherited coagulation abnormalities, no informed consent.

**Intervention and comparator:**

The intervention group will receive the anticoagulant bivalirudin intravenously with a target aPTT of 45-70 sec for three days while the control group will stay on the standard treatment with low-molecular-weight heparins /unfractionated heparin subcutaneously (see scheme in Additional file 1).

All other treatment will be unchanged and left to the attending physicians.

**Main outcomes:**

As a surrogate parameter for clinical improvement and primary outcome we will use the PaO2/FiO2 (P/F) ratio.

**Randomisation:**

After inclusion, the patients will be randomized using a closed envelope method into the conventional treatment group, which uses the standard strategy and the experimental group which receives anticoagulation treatment with bivalirudin using an allocation ratio of 1:1.

**Blinding (masking):**

Due to logistical and safety reasons (assessment of aPTT to titrate the study drug) only the data-analyst will be blinded to the groups.

**Numbers to be randomised (sample size):**

We performed a sample size calculation and assumed the data for P/F ratio (according to literature) is normally distributed and used the mean which would be: 160 and SD is 80. We expect the treatment will improve this by 30%. In order to reach a power of 80% we would need 44 patients per group (in total 88 patients). Taking approximately 10% of dropout into account we will include 100 patients (50 in each group).

**Trial Status:**

The local registration number is MRC-05-082 with the protocol version number 2. The date of approval is 18th June 2020. Recruitment started on 28^th^ June and is expected to end in November 2020.

**Trial registration:**

The protocol is registered before starting subject recruitment under the title: “Anticoagulation in patients suffering from COVID-19 disease. The ANTI-CO Trial” in ClinicalTrials.org with the registration number: NCT04445935. Registered on 24 June 2020.

**Full protocol:**

The full protocol is attached as an additional file, accessible from the Trials website (Additional file 2). In the interest in expediting dissemination of this material, the familiar formatting has been eliminated; this Letter serves as a summary of the key elements of the full protocol.

## Supplementary information

**Additional file 1.**

**Additional file 2.** Full Study Protocol.

## Data Availability

The final dataset will be available to the research-team, to the local authorities and upon reasonable request to others after agreement of the local IRB. All data are anonymised and stored safely for five years according to local law.

