# Supplementary material for: Anticoagulation in critically ill patients on mechanical ventilation suffering from COVID-19 disease, The ANTI-CO trial: A structured summary of a study protocol for a randomised controlled trial
Source: Trials. 2020 Sep 7;21:769. doi: 10.1186/s13063-020-04689-1 (PMC7476433; doi:10.1186/s13063-020-04689-1)
Supplement: Supplementary file 1 — Additional file 1. [file 13063_2020_4689_MOESM1_ESM.zip › Bivalirudin_HIT protocolR0.pdf]

Hamad Medical Corporation
مؤسسة حمد الطبية
HEALTH • EDUCATION • RESEARCH صحة • تعليم • بحوث

Patient sticker

Heparin induced thrombocytopenia protocol:

Bivalirudin

Patient weight Kg
Patient height cm

First order should be complete by MD/ MICU-specialist
Stop all heparin or low molecular weight heparin, including flash or locks
Label all the IV sites or catheters as “**NO HEPARIN**”
Order CBC without differential daily.
Base line for aPTT prior to infusion.
STAT aPTT 2 hours after the start of the continuous infusion and 2 hours after any rate change.

Initial dose should be based on the calculated CrCl using Cockcroft–Gault equation as following;

**Initial maintenance infusion (250mg/250ml NS or D5W)**	
CrCl (ml/min)	Dose(Based on actual body weight)
>60	0.13mg/kg/hr
30-60	0.08mg/kg/hr
<30 or CRRT	0.05 mg/kg/hr
IHD	0.07mg/kg/hr

Document the initial, the rate
Document aPTT lab draw and result on the HIT protocol flow record
Adjust rate of infusion based upon Bivalirudin dose adjustment instruction

**BIVALIRUDIN Dose adjustment instructions (Use a standard concentration 1mg/1ml)**	
aPPT (seconds)	Dose adjustment/ monitoring
Greater than 75	Stop infusion for 1 hour and then restart at 50% slower rate<br>New rate=current rate/2) then draw aPTT after 2 hours after each rate change
45-75	Continue at current rate.
Less than 45	Increase infusion rate by 20% (new rate= current rate X 1.2)<br>(remember –Draw aPTT 2 hours after each rate change

- If any two sequential aPTT higher than 75 second, hold the infusion and inform clinical pharmacist and MD on-call
- Don’t start warfarin until Platelet> 150 000/mm^3 . Not more than **5 mg** starting dose.
- Minimum 5 days of overlap with Bivalirudin and warfarin.
