# Supplementary material for: Anticoagulation in critically ill patients on mechanical ventilation suffering from COVID-19 disease, The ANTI-CO trial: A structured summary of a study protocol for a randomised controlled trial
Source: Trials. 2020 Sep 7;21:769. doi: 10.1186/s13063-020-04689-1 (PMC7476433; doi:10.1186/s13063-020-04689-1)
Supplement: Supplementary file 1 — Additional file 1. [file 13063_2020_4689_MOESM1_ESM.zip › standard anticoagulation HMGHR0.pdf]

Anticoagulation in COVID 19

D-Dimer	Weight	Dose	Monitoring
**< 1.2 mg/L ,**<br><br>**No suspected VTE**	**< 90 kg**	Enoxaparin **40** mg SC **OD** ,<br>If CrCl < 30 ml/min Dalteparin **5000** units SC **OD**	**Anti-Xa** target **0.2 - 0.4**<br>(4 hrs after the 3rd dose)
	**90 - 120 kg**	Enoxaparin **40** mg SC **OD** ,<br>If CrCl < 30 ml/min Dalteparin **5000** units SC **OD**	
	**> 120 kg**	Enoxaparin **60** mg SC **OD** ,<br>If CrCl < 30 ml/min Dalteparin **7500** units SC **OD**	
**> 1.2 mg/L ,**<br><br>**No suspected VTE ,**<br><br>**No riskof bleeding (Low Fibrinogen , Low Plt)**	**< 90 kg**	Enoxaparin **60** mg SC **OD** ,<br>If CrCl < 30 ml/min Dalteparin **7500** units SC **OD**	**Anti-Xa** target **0.4 - 0.6**<br>(4 hrs after the 3rd dose)
	**90 - 120 kg**	Enoxaparin **40** mg SC **BID** ,<br>If CrCl <30 ml/min Dalteparin **10000** units SC **OD**	
	**> 120 kg**	Enoxaparin **60** mg SC **BID** ,<br>If CrCl < 30 ml/min Dalteparin **7500** units SC **BID**	
**> 1.2 mg/L ,**<br><br>**Suspected VTE ,**<br><br>**Or Unexplained increase in Oxygen requirement ,**<br><br>**Or Organ failure**		Enoxaparin **1 mg/kg** SC **BID** ,<br>If CrCl < 30 ml/min Dalteparin **100 units/kg** SC **BID**	**Anti-Xa** target **0.6 - 1.0**<br>(4 hrs after the 3rd dose)
