# Supplementary material for: Anticoagulation in critically ill patients on mechanical ventilation suffering from COVID-19 disease, The ANTI-CO trial: A structured summary of a study protocol for a randomised controlled trial
Source: Trials. 2020 Sep 7;21:769. doi: 10.1186/s13063-020-04689-1 (PMC7476433; doi:10.1186/s13063-020-04689-1)
Supplement: Supplementary file 2 — Additional file 2. Full Study Protocol. [file 13063_2020_4689_MOESM2_ESM.pdf]

# Anticoagulation in patients suffering from COVID-19 disease

## The ANTI-CO trial

**Participants (all enrolled in ABHATH):**

Dr. Marcus Lance, Senior Consultant, Anesthesia, SICU

Dr. Stefan Roehrig, Senior consultant, SICU

Dr. Nadir Kharma, Senior Consultant, MICU

Dr. Moustafa Sayed Elshafei, Consultant MICU

Dr. Hisham Zighlam, Senior Consultant ID

Dr. Ingi Mohamed Abbas Elsaid

Dr. Salma Faisal Eltayeb Mustafa

Dr. Osamah Smain

Dr. Asjad Aldabi

Dr. Dema Osman

**Synopsis:**

Patients with COVID-19 associated ARDS and mechanical ventilation have a high mortality. Part of the disease is an activation of the coagulation system which seems to contribute to clotformation in the pulmonary bloodstream. Recently we implemented an algorithm applying higher doses of heparins (LMWH). However, this approach could not inhibit clotformation enough. Bivalirudin could prevent clotformation better and support dissolving existing clots.

Therefore, we want to compare 50 patients with the standard treatment with 50 patients under bivalirudin treatment which we normally apply in patients with a HIT-syndrome.

Our primary outcome measure is oxygenation reflected as P/F ratio.



HMC-IRB,MRC-05-082,18Jun20-17Jun21

**Abbreviations and Acronyms**

List abbreviations, acronyms and terms of reference used in the protocol; provide definitions for each as needed

COVID-19-Coronavirus Disease-2019

SARS-CoV-2: severe acute respiratory syndrome coronavirus 2

ARDS-acute respiratory distress syndrome

ICU-intensive care unit

HGH-Hamad General Hospital

HMGH: Hazm Mebaireek General Hospital

IL-6-Interleukin 6

aPTT-activated partial thromboplastin time

INR- International Normalized Ratio

HIT- Heparin induced thrombocytopenia

antiXa-anti Factor Xa level

AT-Antithrombin

LMWH-Low molecular weight heparin

ETCO2- end tidal CO2

PaO2/FiO2- arterial partial oxygen pressure/ inhaled oxygen fraction

DVT-deep vein thrombosis



HMC-IRB,MRC-05-082,18Jun20-17Jun21

**Introduction:**

The pandemic of COVID-19, a newly upcoming viral disease caused by SARS-CoV-2, puts the whole worlds health system under pressure.

Patients suffering from this disease mainly develop respiratory symptoms, which can lead to severe acute respiratory distress syndrome (ARDS) necessitating ICU admission in 10-20% of the cases admitted to hospital. In addition to these symptoms, patients show lymphopenia, cardiac symptoms and altered coagulation profiles. Although those patients are treated in the ICU the mortality there is more than 20% due to multiorgan failure [1, 2].

One of the recent insights in this disease is its effect on the coagulation system. Meanwhile we know that the coagulation system gets activated. Furthermore, it seems that clot formation takes place in the pulmonary micro-vasculature which could contribute to the widely observed gas-exchange impairment. Therefore, many centers apply empiric anticoagulation for their COVID-patients. At the moment, we at HMC, also apply an empirical anticoagulation protocol as our standard approach (see attachment 1). However, this is a symptomatic treatment without good proof.

Bivalirudin is a well-known agent which is used in HMC for cases in which anticoagulation is needed, but a contraindication for heparin exists (i.e. HIT syndrome). This drug has an interesting pharmacologic profile. It acts independent of antithrombin (AT), the physiological compound which enhances heparin effects under normal circumstances. This lack of dependence on AT, makes Bivalirudin an attractive choice in light of the contradictory reports on the levels of AT during COVID infection. If AT levels are decreased during the infection, heparin (as well as LMWH) cannot work efficiently, which would render our treatment less effective. Luckily, bivalirudin acts without the support of AT, so we could bypass this problem. In addition, bivalirudin has some fibrinolytic activities. It inhibits clot-bound thrombin which as a result destabilizes the clot rendering it more susceptible to thrombolysis. This property partially supports the dissolving of clots and could support re-opening the pulmonary microcirculation. [3,4]

**Objectives:**

To prove the positive effect of anticoagulation with bivalirudin intravenously on gas-exchange in patients with COVID-19 and respiratory failure on invasive mechanical ventilation.

**Primary objective:**

As a surrogate parameter for clinical improvement we will use the PaO2/FiO2 ratio.



**HMC-IRB,MRC-05-082,18Jun20-17Jun21**

**Secondary objectives:**

Clinically parameters (e.g. time to extubation, ventilator settings)

Coagulation function (assessed by laboratory parameters)

Kidney function (assessed by laboratory parameters and urine output)

Liver function (assessed by laboratory parameters)

Immune function (assessed by laboratory parameters)

Cardiac function (assessed by echocardiography and clinical parameters)

Deadspace fraction estimates (from ETCO2 and PaCO2)

30 day and 90 day mortality (hypothesis generating)

**Methods:**

All patients coming to admission to the ICU for COVID associated respiratory distress and in need for mechanical ventilation according to clinical decision are screened for eligibility.

Inclusion criteria: all adult patients admitted to the ICU who are COVID positive tested and in need for mechanical ventilation are eligible for inclusion. Upon crossing the limit of D-dimers (1.2 mg/L) these patients would be standardly treated with an increased does of anticoagulant. This would be the start of randomization.

Exclusion criteria: pregnancy, allergic to the drug, inherited coagulation abnormalities, no informed consent.

After inclusion the patients will be randomized using a closed envelope method into the conventional treatment group (see attached protocol), which uses the HMGH-approved strategy and the experimental group which receives anticoagulation treatment with bivalirudin (see attached HIT-protocol).

As a safety measure we will monitor and titrate all drugs according to the aPTT and/or antiXa (according to the protocols-see attachment).



HMC-IRB,MRC-05-082,18Jun20-17Jun21

**Data collection:**

We will collect demographic data (age, sex, BMI), co-morbidities and clinical data (need for special intervention e.g. proning).

Next to the vital parameters (blood pressure, heart frequency and body temperature) the oxygenation parameters and ETCO2 will be collected.

The standard coagulation parameters (aPTT, INR, platelet count, antiXa) will be collected. In addition, we monitor fibrinogen levels and D-dimers (both are associated with mortality) and AT.

Kidney-function parameters (creatinine, urea), liver function tests and whole blood counts and immune-parameters like interleukin 6 (IL-6) will be recorded.

Cardiac enzymes will be collected (troponin, BNP) and echocardiography will be evaluated and reported.

All of these parameters are reported routinely on daily base for clinical purpose.

**Statistical analysis:**

Power-calculation: Grasselli et al. found that the median PF ratio was 160 with (IQR114-220) in their cohort of 1591 patients.[2] To perform sample size calculation, we assume data is normally distributed and set the median equal to mean (mean=median and SD = IQR/1.35). So, the mean would be: 160 and SD is 80. We expect the treatment will improve this by 30%. In order to reach a power of 80% we would need 44 patients per group (in total 88 patients). Taking approximately 10% of exclusion into account we will include 100 patients (50 in each group).

Data analysis: Categorical data will be presented as number and percentage, while interval data will be presented by median and interquartile range (IQR). Normally distributed data will be analyzed by using two-tailed unpaired Students t-test. Continuous variables with skewed distribution will be analyzed using Mann-Whitney U testing and dichotomous variables by means of Fisher's exact test. A p-value <0.05 is considered significant. All data analyses will be done using SPSS version v23 (IBM Corp, Armonk NY, USA). Graphs will be constructed using GraphPad Prism (GraphPad Prism version 5.0a for Windows, GraphPad Software, San Diego CA, USA).

**Confidentiality**

We will maintain privacy for the subject throughout data collection by carrying out data collection in private rooms (ICU-rooms). Data confidentiality will be maintained by the use of study IDs rather than any identifying data.

All data will be entered into a secure database, which is password protected with restricted access, only assigned by the research team. Each subject will be assigned an alphanumeric study ID, to ensure data confidentiality. The link between the identifier and the study code will be

deleted at the end of the study and the anonymized data set will be kept for at least 5 years after study completion per MRC policy.

**Subject Withdrawal/ Withdrawal of Consent**

Subjects can withdraw from the study at any point and this will not be held against them. They will be informed during the consent process of this and will be asked to contact the research team so that the investigator can withdraw them from the study. If a subject should withdraw, the data and samples collected will be destroyed, unless they have already been analyzed or processed or coded.

**Data Safety and Monitoring Board (DSMB) plan**

This study is an interventional trial using anticoagulants. The anticoagulant used (bivalirudin) principally is registered and FDA approved for the use as a direct thrombin inhibitor in adults undergoing percutaneous coronary interventions and in patients with heparin induced thrombocytopenia. As with all medication this medication might also show side effects. Next to allergic reaction the most prominent could be bleeding. As a bleeding might be a severe side effect, data after the first 5 patients will be reviewed by the DSMB.

If the major bleeding rate exceeds the numbers reported in literature (3.1%) we will be forced to stop the study prematurely due to safety concerns.

**Adverse Event Reporting**

Although the research drug (bivalirudin) is an old drug which is approved and used widely we will follow and report all adverse effects to ensure safety for the patients.

**Ethical considerations:**

The members of the research team act according to the GCP guidelines. All research is done under recognition of the Helsinki declaration and under full adherence to the MoPH regulations in Qatar.

**References:**

1. Onder G, et al. Case-Fatality Rate and Characteristics of Patients Dying in Relation to COVID-19 in Italy. JAMA. 2020 Mar 23. doi: 10.1001/jama.2020.4683.
2. Grasselli G, Zangrillo A, Zanella A, et al. Baseline Characteristics and Outcomes of 1591 Patients Infected With SARS-CoV-2 Admitted to ICUs of the Lombardy Region, Italy. JAMA. 2020;323(16):1574–1581. doi:10.1001/jama.2020.5394



HMC-IRB,MRC-05-082,18Jun20-17Jun21

3. Andreas Koster, David Faraoni, Jerrold H. Levy; Argatroban and Bivalirudin for Perioperative Anticoagulation in Cardiac Surgery. Anesthesiology 2018;128(2):390-400. doi: https://doi.org/10.1097/ALN.0000000000001976.
4. He, S, Blombäck, M, Bark, N, Johnsson, H, Wallén, NH . The direct thrombin inhibitors (argatroban, bivalirudin and lepirudin) and the indirect Xa-inhibitor (danaparoid) increase fibrin network porosity and thus facilitate fibrinolysis. Thromb Haemost 2010; 103:1076–84
